# Box–Behnken Design: Optimization of Proanthocyanidin-Loaded Transferosomes as an Effective Therapeutic Approach for Osteoarthritis

**DOI:** 10.3390/nano12172954

**Published:** 2022-08-26

**Authors:** Neelakandan Tamilarasan, Begum M. Yasmin, Posina Anitha, Hani Umme, Wan Hee Cheng, Sellapan Mohan, Sundarapandian Ramkanth, Ashok Kumar Janakiraman

**Affiliations:** 1 Department of Pharmaceutics, Karpagam College of Pharmacy, Coimbatore 641032, TN, India; 2 Department of Pharmaceutics, King Khalid University, Abha 62529, Saudi Arabia; 3 Department of Pharmaceutics, Annamacharya College of Pharmacy, Rajampet 516126, AP, India; 4 Faculty of Health and Life Sciences, INTI International University, Nilai 71800, Negeri Sembilan, Malaysia; 5 Faculty of Pharmaceutical Sciences, UCSI University, Cheras, Kuala Lumpur 56000, Malaysia

**Keywords:** Box–Behnken, entrapment efficiency, proanthocyanidin, transferosomes

## Abstract

Transferosomes are one of the vesicular carriers that have received extensive research and attention recently because of their capacity to get beyond the barriers posed by the stratum corneum to penetration. The intent of the current study is to optimize and evaluate proanthocyanidin (PAC) containing transferosomal transdermal gels. PAC-containing transferosomes were prepared using the film hydration method and then loaded into a 4% methylcellulose gel. A 2^3^ Box–Behnken design was used to optimize the PAC-loaded transferosomal gel, where the effects of phospholipid 90 G (X1), Tween 80 (X2), and sonication time (X3) were evaluated. The formulation factors, such as the drug entrapment efficiency percentage (PEE) and *in vitro* drug release, were characterized. A PEE of 78.29 ± 1.43% and a drug release in vitro at 6 h of 24.2 ± 1.25% were obtained. The optimized transferosomal-loaded proanthocyanidin (OTP) formulation penetrated the porcine skin at an excellent rate (0.123 ± 0.0067 mg/cm^2^/h). Stability tests were conducted for OTP to predict the effects of various temperature conditions on the physical appearance, drug content, and PEE for periods of 15, 30, and 45 days. Finally, this transferosomal system for transdermal PAC delivery may be a suitable alternative to the conventional treatment for osteoarthritis.

## 1. Introduction

The German company IDEA AG has registered the word “transferosome” as a trademark, which is derived from the Greek word “soma”, meaning “body”, and the Latin word “transferre”, meaning “to transport across” [1]. Lipid-based transferosomes are stiff lipid bilayers (liposomes) or non-ionic surfactant monolayer vesicles (niosomes) that are flexible, ultradeformable, and stress-responsive [2]. While the drug delivery systems such as nanoparticles, liposomes, and niosomes are applied to the skin, they typically can only penetrate the outermost layers of the stratum corneum (SC), accumulating in the epidermal layer, but are not able to penetrate deeper layers of the skin, such as the dermis, or reach systemic levels that are effective. Liposomes are commonly preferred drug delivery methods for cutaneous applications. According to published papers, small unilamellar liposomes have a higher skin penetration capability than bigger ones [3].

Despite being more durable and impervious to modifications in osmolarity than regular liposomes, niosomes’ piercing capacity has been linked to lower fluxes through the SC [4]. When likened to liposomes and niosomes in liquid media, transferosomes have asserted the greatest colloidal stability in the form of zeta potential [5]. Transferosomes have also displayed excellent colloidal stability (a sign of no aggregation) of up to three months at both 4 and 25 °C, whereas niosomes and liposomes have exhibited worse physical stability with a higher propensity for aggregation [6,7]. This fact explains why, to extend the shelf-life of the product, the majority of marketed liposomal formulations are in freeze-dried powders [8].

Transferosomes are formulated by modifying the lipid elements of liposomes with an edge activator (EA) or surfactant [9]. The resulting transferosomes are more elastic than conventional liposomes due to the presence of the surfactant, which improves their capacity to penetrate via tiny pores and reduces the risk of bilayer damage that could happen with hard liposomes [10,11]. Transferosomes can overcome the barrier to penetration by squeezing along the stratum’s intercellular sealing lipid. After being applied to the skin corneum, transferosomes can follow the innate water gradient through the epidermis due to the flexible membranes’ reduced chance of total vesicle rupture [12].

A class of polyphenolic substances known as flavonoids has been shown to significantly improve human health [13]. Tannins are formed when some flavonoids, such as the flavan-3-ols catechin and epicatechin, polymerize. Proanthocyanidins (PACs) are another name for the condensed tannins [14,15], which act as a protection against biotic and abiotic stresses; they are found in the seeds, fruits, flowers, nuts, and bark of many plants. The plants are shielded from viruses and predators by their astringency. They are byproducts of the flavonoid biosynthesis pathway that are oligomeric and polymeric. Catechin and epicatechin are the PAC structural constituents [16].

One of the most prevalent joint disorders, with a significant impact on patients’ daily lives, is osteoarthritis (OA) [17]. It affects approximately 630 million people worldwide, with males and females being more likely to contract it at varying rates [18,19]. Among the main risk factors for OA are age, sex, genetics, lifestyle, and obesity [20]. PACs from grape seeds may promote lymphocyte differentiation, increase lysosomal enzyme activity, and improve the phagocytic capacity of peritoneal macrophages. TNF (tumor necrosis factor) production was encouraged [21]. PACs have been shown to reduce inflammation in the airways of mouse asthma models by reducing inflammatory cells, Th2 (T helper) cytokines, and serum IgE levels [22]. In an osteoarthritis rat model, grape seed PAC extract decreased chondrocyte loss and proteoglycans [23].

The principle of the response surface method (RSM) is based on the generation of polynomial mathematical relationships and the mapping of responses over an experimental domain to select an optimum formulation. The Box–Behnken design (BBD) is a divergent type of RSM design available for statistical optimization of formulations. This design offers a far more effective method than the conventional techniques of dosage form optimization because it involves many trial runs and less time [24].

In the current study, we use a Box–Behnken design to develop and optimize a PAC-containing transferosomal gel for better transdermal penetration. Additionally, we investigate the permeation efficiency of the optimized transferosomal-loaded proanthocyanidin (OTP) gel.

## 2. Materials and Methods

### 2.1. Materials

The proanthocyanidin (PAC) was a gifted sample from Verax Life Science, Himachal Pradesh, India. Phospholipid 90 G (PC) was purchased from Lipoids Switzerland. Tween 80, sodium hydroxide pellets, and potassium dihydrogen orthophosphate were purchased from Hi-media Pvt Ltd., Mumbai, India. Ethanol and chloroform were procured from Changshu Hongshu Great Chemical Co Ltd., Mumbai, India. All the chemical compounds were of analytic grade (AR).

### 2.2. Compatibility Evaluation Using Fourier Transform Infra-Red Spectroscopy (FTIR)

To provide the optimum interaction, the drug and excipient ratio in the formulation of transferosomes was studied at a 1:1 proportion. Additionally, the interaction between the drug and excipient was verified by FTIR (Shimadzu IR Affinity, Kyoto, Japan) in the scanning range of 4000–400 cm^−1^ using the KBr technique [25].

### 2.3. Preparation and Optimization of PAC-Loaded Transferosomes

PAC-loaded transferosomes, adopting the thin film hydration approach, were formulated. In brief, PACs, lipids (phospholipid 90 G), and Tween 80 were dissolved in 40 mL of chloroform; when the chloroform evaporated at 120 rpm and 40 °C, a thin film was created in the rotary evaporator. To remove the extra solvent, a thin layer of lipids was dried for 10 min at 50 °C and under vacuum overnight. The dried thin film was then hydrated with phosphate-buffered saline (PBS) pH 7.4 for 30 min while being shaken at a temperature that was 10 °C above the amphiphiles phase transition temperature (T_c_). The dispersion was allowed to fully hydrate for 4 h at room temperature, after which it was subjected to sonication for the predetermined number of minutes, as indicated in the design, in order to break the multilamellar liposomes [25] and transfer them to a 4% w/v methylcellulose gel.

### 2.4. Experimental Design

Phospholipid concentration (mg) (X1), Tween 80 (mL) (X2), and sonication duration (min) (X3) were the three main independent variables that were adjusted at two levels, a low level (−1) and a high level (+1), in a three-factor, two-level factorial design (2^3^) for the optimization approach. Following preliminary tests, the values of two coded levels of three components were presumptive; they are displayed in Table 1. The dependent variables were the percentage entrapment efficiency (PEE) (Y1) and *in vitro* diffusion at 6 h (Y2) of prepared transferosomal gels containing PAC through skin. Design Expert 11 software was employed for the creation and assessment of the statistical experimental design. Table 2 displays the design’s response matrix, which includes the permeation flux as a response.

### 2.5. Determination of Percentage Entrapment Efficiency (PEE)

Transferosomal suspensions were ultracentrifuged for 30 min at 20,000 rpm and 10 °C. A UV–visible spectrophotometer (UV 1800 Shimadzu, Kyoto, Japan) was used to detect absorbance at 278 nm after centrifugation, and 1 mL of the supernatant was diluted by the addition of 9 mL of phosphate saline buffer (pH 7.4) [26]. The efficiency of drug entrapment was determined [27] as follows:

(1) $$PEE=\frac{W_{T}-W_{F}}{W_{T}}\times 100$$

where *PEE* is the drug entrapment efficiency, *W_T_* is the total quantity of drug in the transferosomal suspensions, and *W_F_* is the free drug in the supernatants.

### 2.6. Size, Polydispersity Index, and Zeta Potential

Transferosomes were assessed for particle size, polydispersity index (PDI), and zeta potential using a dynamic light scattering device (Zetasizer Nano; Malvern Instruments Ltd., Malvern, UK). For estimation, 1 mL of the formulation was introduced to the analyzer in the see-through Malvern zeta potential cuvette [28].

### 2.7. Scanning Electron Microscopy (SEM)

SEM was employed for the visualization of the vesicle morphology. A glass slide was covered with one drop of transferosomal preparation, which was then spread out and left to dry. The item was coated with gold using a cool spit coater after it had dried and was then visualized with a scanning electron microscope at a voltage of 10 KV [29,30].

### 2.8. Transmission Electron Microscopy (TEM)

TEM analysis was used to examine the surface morphology of manufactured transferosomes (Jeol, JEM-1010, Tokyo, Japan). Before being mounted into the microscope, a silicon wafer was coated with gold and air-dried at room temperature with a diluted suspension of transferosomes (transferosomes:water, 1:5) in Milli-Q water. The image was taken at a 5 kV accelerating voltage [31].

### 2.9. Drug Content 

The vesicles were lysed by sonication for 15 min with 10 mL of methanol and 1 g of the transferosome gel formulation. Later, a centrifuge tube containing this solution was filled, and it was spun at 2000 rpm for one hour. A liquid supernatant was discarded, and dilution was applied as necessary. A UV spectrophotometer (UV 1800 Shimadzu) set to 278 nm was used to measure absorbance [32].

(2) $$\%\;\mathrm{Drug}\;\mathrm{Content}=\frac{\mathrm{Amount}\;\mathrm{of}\;\mathrm{drug}\;\mathrm{obtained}\;\mathrm{after}\;\mathrm{centrifugation}}{\mathrm{Amount}\;\mathrm{of}\;\mathrm{drug}\;\mathrm{taken}}\times 100$$

### 2.10. In Vitro Drug Diffusion Studies

Franz diffusion cells with a diffusional area of 3.14 cm^2^ were used to conduct *in vitro* drug diffusion tests at a time interval of 1, 2, 4, and 6 h for all the formulations. In between the donor and receptor compartments, an egg layer [33] was placed. The formulation was applied to egg film. The phosphate buffer pH 7.4 in the receptor compartment was continuously mixed by a remotely controlled Teflon-coated magnetic bead. To imitate physiological conditions, the temperature of the cell was maintained at 37 ± 1 °C. A UV spectrophotometric approach was used to measure the concentration of the PAC in aliquots (1 mL each) throughout the course of 6 h at regular intervals. Similarly, the OTP was estimated for *in vitro* diffusion studies for 24 h at periodic time intervals. The results were fitted to various kinetic studies [34]. The diffusion study was carried out in triplicate.

#### Method for Egg Membrane Preparation

Egg shells had their contents taken out before being immersed for 30 min in diluted hydrochloric acid. The egg membrane was carefully detached and thoroughly cleaned with distilled water [35,36].

### 2.11. Preparation of Skin for In Vitro Skin Permeation Study

Porcine ear skin was purchased from the slaughterhouse within an hour after the animal was killed, prepared in accordance with the literature, and used in all permeation studies [37]. After using an animal hair clipper to remove the hair from the upper half of the skin surface, the entire thickness of the skin was gathered. By using a surgical scalpel, the fatty layer that was adherent to the dermis side was removed. The skin was then wrapped with aluminum foil after being cleaned with deionized water. The skin samples were used within a week after being kept at 20 °C.

#### *Ex Vivo* Skin Permeation Study

Utilizing a Franz diffusion cell, *ex vivo* skin permeation tests of the improved formulation were performed. The cell has two compartments: the donor and the receptor, each with a 1.43 cm^2^ diffusion area. The donor compartment was exposed to the atmosphere and had a top opening. The stratum corneum of the resected pig ear skin was positioned to be donor-compartment-facing and clamped between the compartments of the diffusion cell. The receptor chambers were filled with the receptor phase, and magnetic stirrer bars were added. As a receptor medium, PBS (pH 7.4) was utilized. To stop any microbial growth, a very small amount of sodium azide (0.0025% *w/v*) was applied [38]. A magnetic stirrer was used to keep the temperature of the entire setup at 37 ± 0.5 °C. To help the skin samples hydrate, the skin sections were originally kept in the Franz cells for 2 h. Following this time frame, 5 mL of the OTP was applied to the skin’s surface. Transferosomal gels were used in the examination of *ex vivo* skin permeability. Over the course of the investigation, 0.5 mL of medium was taken out of the receptor compartment at regular intervals and refilled with the same influx of buffer. Utilizing a UV–visible spectrophotometer, the amount of drug that had diffused was determined by scanning the absorbance at 278 nm.

### 2.12. Physical Stability of the Transferosomes

Stability tests were carried out for 45 days at various temperatures to ascertain the formulation transferosome vesicles’ capacity to retain drugs under changes in temperature and relative humidity (RH). According to the recommendations of the International Conference on Harmonization (ICH), OTP was placed in a sealed vial and kept at 4 °C and room temperature for 15, 30, and 45 days. The physical appearance, drug content, and PEE were examined after samples were removed [39].

## 3. Results and Discussion

In the present investigation, to get around the primary problems with its oral distribution, PAC-loaded transferosomes were created, developed, and tested for their potential to be delivered transdermally. Specifically, non-toxic and biocompatible surfactant namely tween with phospholipid were used to study the transferosome gels.

### 3.1. Compatibility Evaluation Using Fourier Transform Infra-Red Spectroscopy (FTIR)

**Figure 1:**
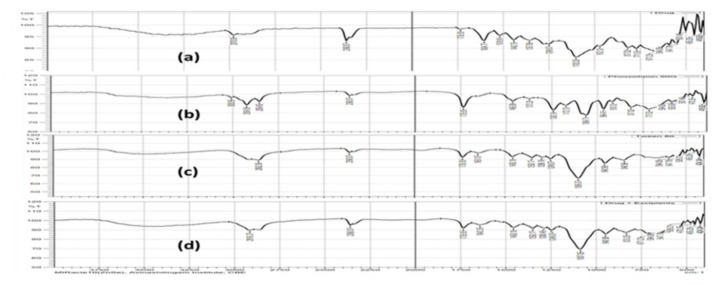
FTIR graphs for the drug polymer compatibility study: (**a**) pure PAC, (**b**) Tween 80, (**c**) phospholipid 90 G, and (**d**) optimized transferosomes-loaded proanthocyanidin.

The FTIR spectra depict the combination of drug, tween 80, phospholipid, cholesterol, blank, and OTP. The characteristic peak in the FTIR spectra of pure PAC drug was found at 2978.09 cm^−1^ (dimer O–H), 1442.75 cm^−1^ (aromatic C–C ring), 1249 cm^−1^ (ester C–O stretch), 1357.89 cm^−1^ (alkanes C–H), and 972 cm^−1^ (=C–H out of plane). Tween 80 showed the characteristic peaks at 2862.36 cm^−1^ (alkanes CH stretch), 1735.77 cm^−1^ (C=O stretch), 1651.07 cm^−1^ (alkenes C=C stretch), 1458.18 cm^−1^ (alkanes CH_2_ and CH_3_). When the physical mixture of drug, Tween 80, and phospholipid 90 G was analyzed, the characteristic peaks of drugs were present in the physical mixture similar to that of an individual drug spectrum, and there were no discernable changes in FTIR spectra, which confirmed the absence of any chemical interactions between them [40,41], as shown in Figure 1.

### 3.2. Preparation and Optimization of PAC-Loaded Transferosomes

**Figure 2:**
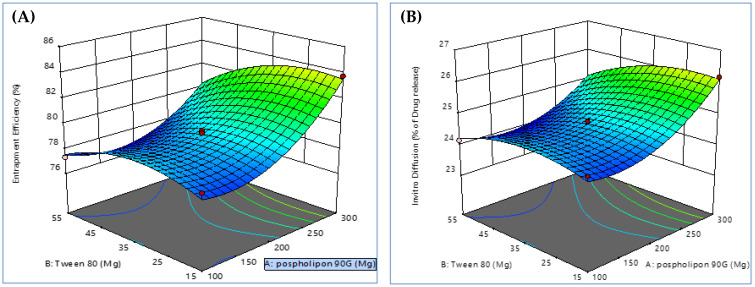
Three-dimensional response surface plot showing the effect of independent variables such as phospholipid concentration (mg) (X1), Tween 80 (mL) (X2), and sonication time (min) (X3) on (**A**) % entrapment efficiency (Y1) and (**B**) *in vitro* diffusion at 6 h (Y2).

**Figure 3:**
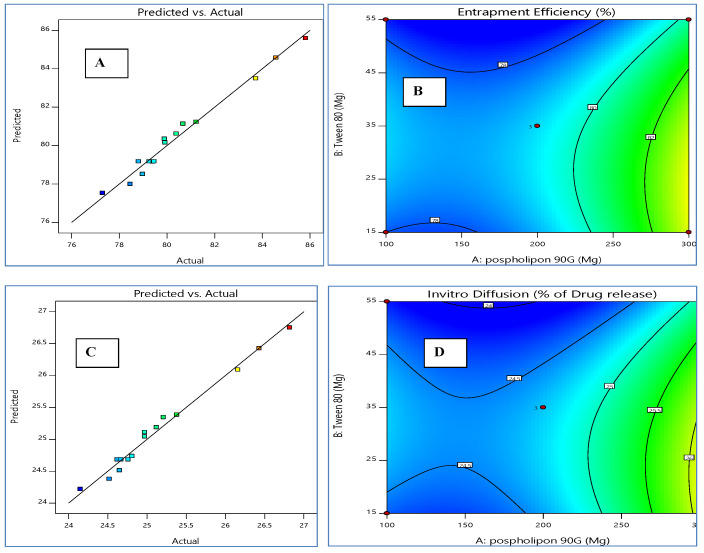
Linear correlation plots (**A**,**C**) between actual and predicted values and the corresponding contour plots (**B**,**D**) for various responses.

Using a Box–Behnken design, a three-factor, two-level, appropriate statistical tool (Design-Expert DX 11 software), and one-way ANOVA at 0.05 levels, a thorough investigation of the effects of process parameters such as phospholipid concentration (mg) (X1), Tween 80 (mL) (X2), and sonication time (min) (X3) and their interactions was the goal of using the experimental design. Since the Box–Behnken design demands minimum runs compared to a central composite design, it was deliberately chosen [42]. A design matrix was produced by the Design Expert software using data from 15 experimental runs. Table 2 displays the outcomes of the 15 formulations that were formulated. The three-dimensional plots for each of the three responses of Y1 and Y2 are displayed in Figure 2. These diagrams are well known for analyzing the interactions between variables and responses as well as for researching the combined effects of two factors on a response. Figure 3 shows a quantitative comparison between the experimental response values that were obtained and those that were anticipated. Table 3 displays the findings of the regression analysis for the various responses. The interactions’ quadratic nature was confirmed by the higher values of the standard error for the coefficient. 

#### 3.2.1. Response 1 (Y1): Effect of Independent Variables on PEE (%)

PEE is the portion of the whole drug (PAC) that is successfully contained within the transferosomes. Phospholipid 90 G has been successfully employed to create vesicular structures resembling liposomes since it is soluble in lipidic or aromatic hydrocarbon carriers but insoluble in polar solvents. Figure 2 displays three-dimensional response surface graphs related to PEE. Figure 3 displays the two-dimensional contour graphs relating to PEE. The three-dimensional response surface plot and the accompanying two-dimensional contour plots show an increase in PEE, with increased Tween 80 up to a certain point before it was discovered to be reduced [43]; that increasing sonication time also increases PEE. Additionally, it was shown that the amount of PEE in transferosomes increased when phospholipid 90 G (X1) levels increased [44].

PEE (Y1) **=** +79.17 + 2.08 X1 − 0.9011 X2 − 0.4071 X3 − 0.6684 X1 X2 − 0.1007 X1 X3 + 0.0825 X2 X3 + 1.97 X1^2^ − 1.29 X2^2^ + 1.86 X3^2^.

The model is suggested to be significant by the model F-value of 31.59. The likelihood of noise producing an F-value this large is only 0.01%. The predicted R^2^ of 0.7613 and the adjusted R^2^ of 0.9516 are reasonably in agreement; therefore, the difference is less than 0.2. The ratio of signal-to-noise is measured with adequate precision. Entrapment efficiency for PAC ranged from 77.29% to 85.81%, with an average of 80.52%. Figure 3 displays the linear correlation plots (C) between the actual and predicted values as well as the associated contour plots (D) for the responses.

#### 3.2.2. Response 2 (Y2): Effect of Independent Variables on *In Vitro* Diffusion at 6 h

The *in vitro* drug diffusion profile at the time period of 6 h for the formulations is shown in Table 2. Due to the lipid’s limited ability to hold substantial amounts of the drug, the free drug may have been disposed of near the surface, which might explain the existence of both free and entrapped drugs. This led to a quick initial release (caused by the presence of the free drug and the drug adsorbed on the surface), followed by a slower sustained release phase (caused by the diffusion of the entrapped drug through the lipid bilayers of the vesicles), which is very effective at sustaining and controlling the release of PAC. These results were in line with those of El Zaafarany et al. [45]. The release rate is significantly impacted by the sonication time.

*In vitro* diffusion at 6 h (Y2) = + 24.68 + 0.6512X1 − 0.2850X2 − 0.1313X3 − 0.2050X1 X2 0.0325 X1 X3 + 0.0200 X2 X3 + 0.6471 X1^2^ − 0.3804 X2^2^ + 0.6071 X3^2^.

As the drug flows through the bilayers of the vesicular structure and results in a decrease in release rate, it is well known that any factor that promotes the production of transferosomes or raises their PEE will impact the release rate [46]. When Tween 80 was used, the diffusion rate was accelerated as the solvent volumetric ratio increased. Additionally, when Tween 80 was used, the release rate was found to be slowed down by increasing the amount of medication administered, which may be related to an increase in PEE [47].

#### 3.2.3. Numerical Point Prediction Method

By using the numerical point prediction optimization method of the software Design Expert 11, the ideal transferosomal formulation was chosen based on the criteria of achieving the acceptable value of the entrapment efficiency of PAC and *in vitro* diffusion at 6 h. The formulation, with phospholipid 90G (175.97 mg), Tween 80 (43.59 mL), and sonication duration (25.79 min), was determined to satisfy the requirements of an OTP formulation with a desirability value of 0.966. Scanning electron microscopy was used to examine the morphology of the optimized formulation. The image showed how the medication became tangled up in the lipid network. It displayed well-known round vesicles of a consistent size with a stable nano-vesicular shape. The entrapment efficiency of PAC was 78.29 ± 1.43%, while *in vitro* diffusion at 6 h for OTP formulation was 24.2 ± 1.25%. Investigative estimates of the PEE of PAC and *in vitro* diffusion at 6 h produced by the OTP were discovered to be in agreement with the predicted values of the PEE of PAC (79.03 ± 1.24%) and *in vitro* diffusion at 6 h (24.67 ± 1.65%) produced by the Design Expert software, indicating that the optimized formulation was plausible and reputable.

### 3.3. Size, Polydispersity Index, and Zeta Potential

**Figure 4:**
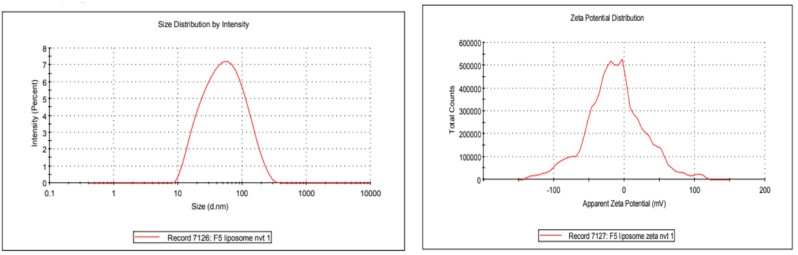
Vesicle size distribution and zeta potential of optimized transferosome-loaded proanthocyanidin.

The outcomes demonstrated that OTP dispersion significantly reduces particle size with a restricted size distribution. It was discovered that the OTP’s average particle size was 65 ± 14.44 nm. Figure 4 shows the distribution of vesicle sizes for the optimized formulation. It was discovered that the improved formulation’s zeta potential was −13.0 mv; prior studies had also revealed a value that was very comparable [48]. The value implies the high-level stability of the optimized formulation. Due to the use of Tween 80, these values indicate that the particles have a neutral charge (a non-ionic surfactant). Tween 80, a surface stearic stabilizer, is used to cover the surface of nanoparticles to stop them from aggregating. The formulation would be stable as a result.

### 3.4. Scanning Electron Microscopy (SEM)

**Figure 5:**
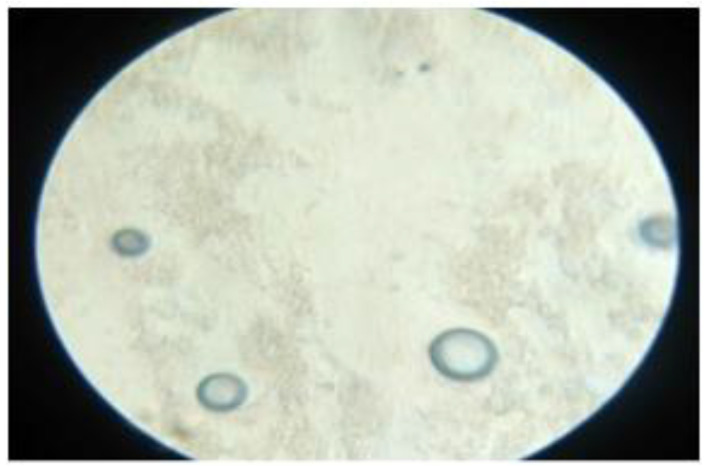
Microscopic vesicular image of optimized transferosome-loaded proanthocyanidin.

**Figure 6:**
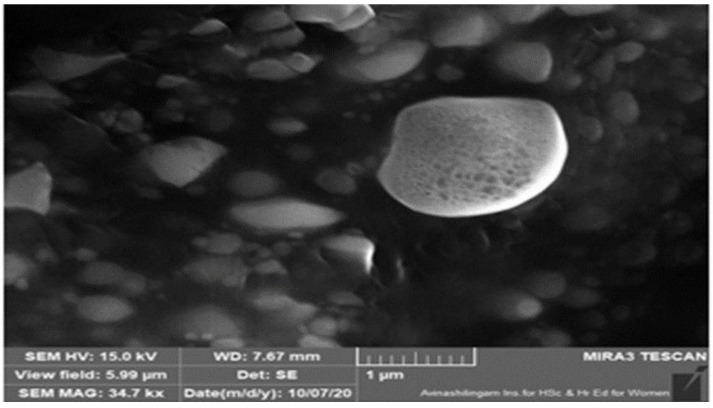
SEM vesicular image of optimized transferosome-loaded proanthocyanidin.

Optical microscopy was used to examine the surface morphology. SEM analysis revealed that the PAC transferosomes (OTP) were found to be merely in spherical forms. Optical microscopical and SEM images are shown in Figure 5 and Figure 6, respectively.

### 3.5. Transmission Electron Microscopy (TEM)

**Figure 7:**
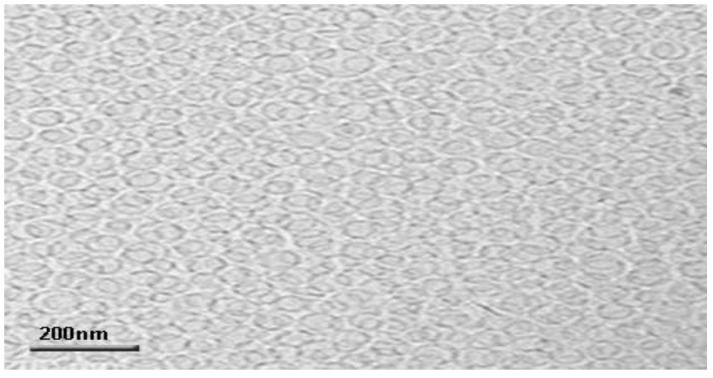
TEM-magnified vesicular image of optimized transferosome-loaded proanthocyanidin.

The formulated OTP was envisioned by TEM; the image is presented in Figure 7. The TEM image witnesses the presence of a multilamellar vesicular structure. Nonetheless, these lamellar vesicles were discovered to be spaced to the core [49].

### 3.6. Drug Content

The results obtained showed 90.28 ± 1.23% drug content in OTP, which shows no degradation of the drug in the process [31].

### 3.7. In Vitro Drug Diffusion Studies

**Figure 8:**
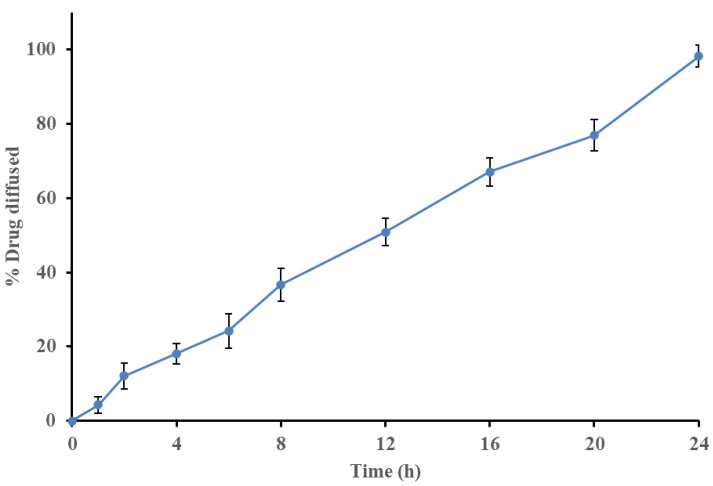
*In vitro* drug permeation graph of optimized transferosome-loaded proanthocyanidin.

*In vitro* drug diffusion of OTP was performed and observed in a UV spectrophotometer at the 278 nm range. The samples were withdrawn at predetermined intervals to determine the percent drug permeation after 24 h. The results were fitted to kinetic studies, such as the order of drug diffusion and Higuchi’s and Peppa’s plots. The OTP shows an R^2^ value of 0.9974 for the *in vitro* diffusion study, which clearly indicates that the formulation obeys a zero-order kinetic pattern. The R^2^ and n-value of Higuchi’s and Peppa’s plots were found to be 0.9424 and 0.9316, respectively, which indicate the release mechanism followed was a diffusion-mediated non-Fickian type. The *in vitro* drug diffusion plots are shown in Figure 8.

### 3.8. Ex Vivo Skin Permeation Study 

**Figure 9:**
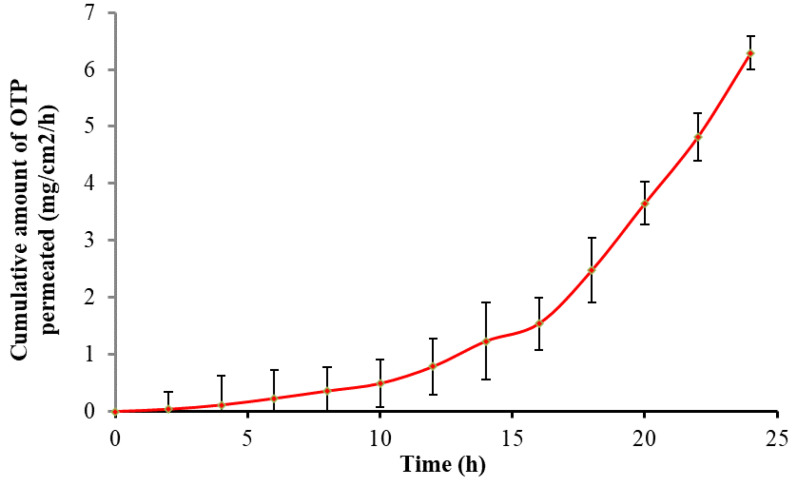
*Ex vivo* drug permeation plot for optimized transferosome-loaded proanthocyanidin.

Franz diffusion cells were used to test the *ex vivo* skin permeation of the designed OTP, utilizing excised pig skin as the permeation membrane. Over the course of 24 h, it was discovered that these transferosomal gels’ *ex vivo* skin penetration of OTP was sustained (Figure 9). These transferosomal gels’ Jss (mg/cm^2^/h) was calculated to be 0.123 ± 0.0067 mg/cm^2^/h. Therefore, the Jss of the transferosomal gel increased as the PEE in transferosomes containing PAC increased. This agreed with earlier studies that found that the transdermal penetration of medication when the deformable vesicles were applied topically was ten times higher than that of conventional vesicles [50]. Deformable vesicles containing sodium deoxycholate may have caused the change in water concentration. It has also been shown that the inclusion of surfactants in the phospholipid vesicles promotes the production of membrane ripples. These ripples also provide the inter-membrane connection location for membrane fusion [37].

### 3.9. Physical Stability of the Transferosomes

The monitoring of the physical appearance, drug content, and PEE under various storage circumstances for a duration of 0, 15, 30, and 45 days was done to determine the stability of the OTP formulation. Throughout the 45 days, no significant changes were seen in any of the parameters when examined at 4 °C and at room temperature (Table 4).

## 4. Conclusions 

The transferosomes were fabricated by loading PAC using the film hydration method; the PAC was incorporated into a 4% w/v methylcellulose gel. The effects of three independent factors such as phospholipid 90 G, Tween 80, and sonication time on PEE and *in vitro* drug release at 6 h were analyzed as responses in the process of identifying the optimized formulation (OTP) using 2^3^ Box–Behnken design-based response surface methodology. A skin permeation flux of 0.123 ± 0.0067 mg/cm^2^/h was demonstrated in *ex vivo* PAC permeation from the OTP through pig skin, which represents a further improvement. Hence, this highly deformable vesicle-based transferosome (OTP) was found to be successive progress in the delivery of PAC through the transdermal route.

## Figures and Tables

**Table 1:** Variables and their levels.

**Independent Variables**	**Factors Level**		
	**Low (−1)**	**Medium (0)**	**High (+1)**
X1 = Phospholipid 90 G (mg)	100	200	300
X2 = Tween 80 (mL)	15	35	55
X3 = Sonication time (mins)	15	25	35
**Dependent variables**	**Constraints**		
Y1 =Entrapment efficiency	Maximize		
Y2 = In vitro diffusion at 6 h	Maximize		

**Table 2:** Factors and responses for all the formulations.

Formulation Code	Factor 1	Factor 2	Factor 3	Response 1	Response 2
	A: Phospholipid 90 G (mg)	B: Tween 80 (mg)	C: Sonication Time (min)	Y_1_: Encapsulation Efficiency (%)	Y_2_: *In Vitro* Diffusion (%)
PAC 1	100	35	15	81.23	25.38
PAC 2	300	35	35	84.57	26.43
PAC 3	300	55	25	79.89	24.97
PAC 4	100	15	25	78.45	24.52
PAC 5	300	35	15	85.81	26.82
PAC 6	200	15	15	80.67	25.21
PAC 7	200	55	35	78.97	24.65
PAC 8	100	35	35	80.39	25.12
PAC 9	200	35	25	79.25	24.76
PAC 10	200	35	25	79.45	24.67
PAC 11	200	35	25	78.80	24.62
PAC 12	300	15	25	83.72	26.16
PAC 13	200	55	15	79.40	24.81
PAC 14	100	55	25	77.29	24.15
PAC 15	200	15	35	79.92	24.97

**Table 3:** Outline of results of regression analysis for responses Y1 and Y2 for fitting to the quadratic model.

Quadratic Model	Lack of Fit *p*-Value	Adjusted R^2^	Predicted R^2^
Response (Y1)	0.2290	0.9516	0.7613
Response (Y2)	0.1267	0.9592	0.7840
**Regression equation of the fitted quadratic model** PEE (Y1) = +79.17 + 2.08X1 − 0.9011X2 − 0.4071X3 − 0.6684X1 X2−0.1007X1 X3 + 0.0825X2 X3 + 1.97X1^2^ − 1.29X2^2^ + 1.86X3^2^ *In vitro* diffusion at 6 h (Y2) = +24.68 + 0.6512X1 − 0.2850X2 − 0.1313X3 − 0.2050X1 X2 − 0.0325 X1 X3 + 0.0200X2 X3 + 0.6471X1^2^ − 0.3804X2^2^ + 0.6071X3^2^			

**Table 4:** Stability studies of formulation-optimized transferosome-loaded proanthocyanidin.

S. No.	Temperature	Physical Appearance				Drug Content (%)				PEE (%)			
		0	15	30	45	0	15	30	45	0	15	30	45
1	At 4 °C	Clear	Clear	Clear	Clear	90.26 ± 0.32	90.06 ± 0.12	90.16 ± 0.43	90.16 ± 0.12	80.78 ± 0.12	80.23 ± 0.15	80.27 ± 0.10	80.34 ± 0.17
2	At Room	Clear	Clear	Clear	Clear	89.99 ± 1.22	89.32 ± 0.43	88.93 ± 0.32	88.23 ± 0.52	79.84 ± 1.35	78.64 ± 0.16	78.94 ± 1.27	78.74 ± 0.25

## Data Availability

Not applicable.
